# Molecular docking analysis of PARγ with compounds from *Ocimum tenuiflorum*

**DOI:** 10.6026/97320630017928

**Published:** 2021-11-30

**Authors:** Ponnulakshmi Rajagopal, Selvaraj Jayaraman, Shazia Fathima JH, Saravanan Radhakrishnan, Patil Ashlesh Laxman, Vijaya Prakash Krishnan Muthaiah, Satyendra Chandra Tripathi, TS Gugapriya, Aaditya Madhusudan Tarnekar, Gayatri Girish Muthiyan, Vishwajit Ravindra Deshmukh, Bharat Ramrao Sontakke, Kirubhanand Chandrashekar

**Affiliations:** 1 Central Research Laboratory, Meenakshi Academy of Higher Education and Research (Deemed to be University), Chennai-600 078, India; 2 Department of Biochemistry, Saveetha Dental College and Hospitals, Saveetha Institute of Medical and Technical Sciences, Saveetha University, Chennai - 600 077, India; 3 Department of Oral and Maxillofacial Pathology, Ragas Dental College and Hospitals, Chennai, India; 4 Department of Biochemistry, Karapaga Vinayaga Institute of Medical and Dental science, Madhuranthagam- 603308, Tamil Nadu, India; 5 Department of Physiology, AIIMS, Nagpur 441108, India; 6 Department of Rehabilitation Science, School of Public Health and Health professions, 633 Kimball, Buffalo NY, 14214-3079; 7 Department of Biochemistry, AIIMS, Nagpur 441108, India; 8 Department of Anatomy, AIIMS, Nagpur 441108, India

**Keywords:** Diabetes, Proliferator - Activated Receptor - γ, *Ocimum tenuiflorum*, Molecular docking

## Abstract

The ligand-activated transcription factor peroxisome proliferator-activated receptor (PPAR) has become a major target for type 2 diabetes. It belongs to the nuclear receptor superfamily, which controls the expression of proteins involved in glucose metabolism, lipid metabolism, adipocyte proliferation and differentiation, and insulin sensitivity. *Ocimum tenuiflorum*, often known as Krishna tulsi, is the most sacred herb in India. It was utilized for a variety of medicinal purposes. Therefore, it is of interest to document the molecular docking analysis data of PARγ modulators from *Ocimum tenuiflorum*. Four of the twenty substances (rosmarinic acid, permethrin, luteolin, and isosakuranetin) have a considerable binding affinity for the PPARγ. These phytochemicals are a source of potential anti-diabetic medicines.

## Background:

Diabetes mellitus has been linked to the pathogenesis of long-term consequences such as atherosclerosis, retinopathy, nephropathy, neuropathy, and microangiopathy, which are produced by increased glucose to fat conversion, sorbitol buildup, and protein glycation [1,2]. Acarbose (a carbohydrate digesting enzyme inhibitor and brush border enzyme inhibitor) and tolbutamide are two synthetic medicines used to treat diabetes mellitus (an agonist of sulfonylurea receptor). While some of these drugs accomplish temporary blood sugar control, they have a number of side effects, including induced hypoglycemia, hepatotoxicity, cardiac failures, and cholestatic jaundice [3,4].

Peroxisome proliferator-activated receptors (PPARs) are a family of nuclear proteins that regulate cellular differentiation and development, as well as metabolic processes such as glucose, lipid, and protein [5] and protect humans from cancer [6]. There are 48 nuclear hormone genomes in PPARs. Weight gain, hypertension, dyslipidemia (expanded blood serum triglycerides; low high-density lipoprotein (HDL) and high low-density lipoprotein (LDL) cholesterol levels), insulin resistance with elevated fasting blood glucose level, and glucose tolerance have all been linked to these factors. The metabolic syndrome also has a role in the foundations of prothrombotic and proinflammatory illness states in humans [7]. In type 2 diabetic individuals, the metabolic syndrome is causing more serious and dangerous cardiovascular issues. In light of this, numerous studies have been conducted on the metabolic syndrome and its associated problems and diseases, including greasy liver, sleep disturbances, cholesterol gallstones, polycystic ovarian condition, asthma, and a variety of cancers [8]. Exceptional efforts have been made to investigate the ability of PPAR-γ modulators to enhance and intensify glucose homeostasis. Research has been done to investigate the PPAR-γ activating potential of a wide range of natural sources found in medicinal plants.

PPAR-γ is a master controller of adipocyte separation and appears to be a vital player in the lipid digestion system and glucose homeostasis, as well as controlling cell growth and tweaking the digestion system and aggravation in resistant cells. It is activated when preadipocytes and adipocytes are separated [9]. According to one study, 119 clinically used plant-derived medications were evaluated, and 74% of them were found to be used for disease indications connected to the traditional use of the medicinal plants from which the compounds were separated [10]. In India, *Ocimum tenuiflorum* (*Ocimum sanctum*) is known as Thulasi/Tulsi and belongs to the Lamiaceae family. It is extensively grown throughout the world and is revered as India's sacred plant. Tulsi, also known as Holy Basil, has been used by Vaishnavas for thousands of years. Fresh leaves of this plant are often used to cure coughs, colds, abdominal pain, skin illnesses, arthritis, severe eye conditions, measles, and diarrhea throughout the Indian subcontinent. Preclinical tests on extracts from several sections of *O. tenuiflorum* revealed anti-fertility, anti-cancer, anti-diabetic, anti-fungal, hepatoprotective, and cardioprotective properties [11]. Therefore, it is of interest to document the molecular docking analysis data of PARγ modulators from *Ocimum tenuiflorum*.

## Materials & Methods:

### Protein preparation:

3D crystal structure of peroxisome proliferator-activated receptor gamma (PPAR-γ) was downloaded from Protein Data Bank (pdb id: 5YCP). Before performing docking studies the protein structure was prepared by removing water molecules present in the crystal structure and co crystallized ligand.

### Ligand Preparation:

20 compounds from *Ocimum tenuiflorum* was selected from literature and the 2d structure of these compounds were downloaded from pubchem database in SDF format. By using online smiles translator the 2d sdf was converted into 3d PDB format. The converted pdb format was load to pyrx for docking analysis (Table 1 - see PDF).

### Molecular docking analysis:

The procedure of docking of ligands with the receptor has been performed using Autodock version 0.8 of pyrx software [12]. The ligand library was created by putting all of the ligands in a pyrx Autodock (Autodock vina) folder [13]. By executing simultaneous docking of numerous ligands against the receptor, the library preparation aids in making an easier comparison research of ligands. Grid batch docking was also carried out. Each docked molecule's outcome is expressed in terms of a final minimum score (Dock score interaction/ docking energy of receptor-ligand).

## Results and Discussion:

PPAR-γ is a ligand-activated transcription factor that controls a variety of metabolic processes, including lipid and glucose homeostasis [14,15]. PPAR- γ promotes glucose homeostasis by controlling the expression of hexokinase and inhibiting G6Pase, as well as modulating the action of insulin, and is a molecular target for medicines to treat a variety of metabolic problems. The glitazone receptor, also known as the peroxisome proliferator-activated receptor gamma (PPAR- γ), has a role in increasing insulin sensitivity and reducing lipotoxicity by promoting the storage of fatty acids in fat cells [16]. To create a binding site for ligands, the PPAR- γ ligand-binding domain is folded into a helical sandwich. It is found near the C-terminus of the PPAR gene. The PPAR- γ ligand-binding domain is made up of roughly 250 amino acids, and complete agonists activate it via hydrogen bond interactions between the Ser289, His323, Tyr473, and His449 residues [17]. As a result, a molecular docking research was conducted using PyRx to explore the binding effectiveness of phytocompounds from *Ocimum tenuiflorum* as a PPAR- γ agonist.

Gbind best value was used to rank the docking outcomes (lowest energy). The lower the Gbind energy, the more stable the conformation established, whereas the higher the Gbind energy, the less stable the complex created. Rosmarinic acid, permethrin, luteolin, and isosakuranetin had binding energies of -8.3, -8.3, -7.3, and -7.1 kcal/mol, respectively. This shows that these chemicals have a high affinity for PPARγ.

The specificity of ligand binding is largely determined by hydrogen bonds. Their essential contribution is directly incorporated into GRID, a computational approach for detecting energetically favorable ligand binding sites on a target molecule with a given structure. Because of the great H-bonding capacities of both H-bond donors and H-bond acceptors, H-bonds play a substantial role in binding affinity. Table 1(see PDF) illustrates the protein residues that interact with phytocompounds via hydrogen bonds. The four chemicals that were chosen established more hydrogen bonds with PPAR. Among the four compounds RosmarinicAcid formed the four hydrogen bonds with amino acids residues of SER-289,HIS-323,HIS-449 and TYR-473 with distance of 1.5,2.4,2.2 and 2.4Å respectively. Permethrin also formed the four hydrogen interaction through SER-289,HIS-323,HIS-449 andTYR-473 with distance of 2.3,2.4,2.4 and 2.2Å. The Luteolin formed two hydrogen with ARG-288,SER-289 at distance of 2.1and 1.3Å. Isosakuranetin formed only one hydrogen interaction with ARG-288 at distance of 2.4Å. The hydrogen bond interaction distance of these compounds occurs below 3Å. It indicates that all the compounds formed the stable interaction with PPARγ. As per literature these compounds showed the interaction in the Ser289, His323, Tyr473, and His449 residues, which was present in the ligand binding domain. Data shows that selected these compounds may acts as potential drug candidate for diabetics after the experimentally validation (Figure 1 - see PDF).

## Conclusion:

Plant-derived chemicals regulate PPAR activity more strongly than other substances. The potential modulators (rosmarinic acid, permethrin, luteolin, and isosakuranetin) revealed a number of molecular interactions with the PPARγ protein's binding site for further consideration.
